# Several Isoforms for Each Subunit Shared by RNA Polymerases are Differentially Expressed in the Cultivated Olive Tree (*Olea europaea* L.)

**DOI:** 10.3389/fmolb.2021.679292

**Published:** 2021-12-20

**Authors:** Isabel Fernández-Parras, Jorge Antolín Ramírez-Tejero, Francisco Luque, Francisco Navarro

**Affiliations:** ^1^ Departamento de Biología Experimental-Genética, Jaén, Spain; ^2^ Centro de Estudios Avanzados en Aceite de Oliva y Olivar, Universidad de Jaén, Jaén, Spain

**Keywords:** RNA polymerases, plants, olive, expression conditions, common subunits

## Abstract

Plants contain five nuclear RNA polymerases, with RNA pols IV and V in addition to conserved eukaryotic RNA pols I, II, and III. These transcriptional complexes share five common subunits, which have been extensively analyzed only in yeasts. By taking advantage of the recently published olive tree cultivar (*Olea europaea* L. cv. Picual) genome, we performed a genome-wide analysis of the genomic composition corresponding to subunits common to RNA pols. The cultivated olive tree genome is quite complex and contains many genes with several copies. We also investigated, for the first time, gene expression patterns for subunits common to RNA pols using RNA-Seq under different economically and biologically relevant conditions for the cultivar “Picual”: tissues/organs, biotic and abiotic stresses, and early development from seeds. Our results demonstrated the existence of a multigene family of subunits common to RNA pols, and a variable number of paralogs for each subunit in the olive cultivar “Picual.” Furthermore, these isoforms display specific and differentiated expression profiles depending on the isoform and growth conditions, which may be relevant for their role in olive tree biology.

## Introduction

Gene expression is a highly regulated process that comprises coordinated steps to ensure appropriate RNA levels and to allow cells to correctly respond and adapt to any situation. Transcription is the most widely studied step in gene expression that is carried out by RNA polymerases (RNA pols). In bacteria, archaea and eukarya RNA pols are heteromultimeric complexes responsible for the specific synthesis of different RNA types (Werner and Grohmann, 2011). Most eukaryotes possess three heteromultimeric RNA polymerases, namely, RNA pol I, RNA pol II, and RNA pol III (also known as RNA pols A, B, and C in plants). While RNA pol I synthesizes the precursor of the three largest rRNAs, RNA pol III synthesizes tRNAs, 5S rRNA, and several short non-translated RNAs. RNA pol II produces all mRNAs and many non-coding RNAs, including miRNA (Kwapisz et al., 2008; Werner et al., 2009; Werner and Grohmann, 2011; Cramer, 2019). Furthermore, plants contain two additional RNA pols—RNA pols IV and V (or RNA pols D and E)—that play roles in epigenetic regulation. They synthesize siRNAs that play roles in transcriptional silencing *via* RNA-directed DNA methylation (RdDM) and also non-coding RNAs with a role in the development and response to environmental changes (Wierzbicki et al., 2008; Tucker et al., 2010; Haag and Pikaard, 2011; Lopez et al., 2011; Ream et al., 2013). Both RNA pols IV and V have evolved as specialized forms of RNA pol II, as demonstrated by mass spectrometry and phylogenetic analyses (Huang et al., 2009; Ream et al., 2009; Tucker et al., 2010; Wang and Ma, 2015).

RNA pols I and III are composed of 14 and 17 subunits, respectively, while RNA pol II contains 12 (Kwapisz et al., 2008; Werner et al., 2009; Werner and Grohmann, 2011; Cramer, 2019). Plant RNA pols IV and V, which have evolved from RNA pol II, are also composed of 12 subunits, some of which are shared with RNA pol II (Wierzbicki et al., 2008; Tucker et al., 2010; Haag and Pikaard, 2011; Lopez et al., 2011; Ream et al., 2013; Wang and Ma, 2015). It has been described that the NRP4 subunit, shared by RNA pols II, IV, and V, is missing in cauliflower RNA pol V. However, this enzyme maintains its role in RNA silencing (Huang et al., 2009).

Eukaryotic RNA pols I, II, and III share five common subunits (Rpb5, Rpb6, Rpb8, Rpb10, and Rpb12) with archaeal homologs (Woychik et al., 1990; Shpakovski et al., 1995; Cuevas-Bermúdez et al., 2017). In plants, several paralogs of subunits common to RNA pols have been identified. Some are shared by several of, or all five, RNA pols, while others are RNA pol–specific. This is the case for subunits NRP5 and NRP6, which are specific or shared only by RNA pol IV and/or V, or even shared by RNA pols II, IV, and V (Wierzbicki et al., 2008; Huang et al., 2009; Tucker et al., 2010; Haag and Pikaard, 2011; Ream et al., 2013; Wang and Ma, 2015). Similarly, Rpb5 and Rpb6 paralogs have been described in trypanosomes (Kelly et al., 2005; Devaux et al., 2006). Subunits common to RNA pols have been extensively analyzed in yeast (Cuevas-Bermúdez et al., 2017), while their role and contribution to transcription are still unclear in plants. Notably, although subunits common to RNA pols must perform similar functions in the corresponding RNA pols, some of these subunits have been described to also play specific roles in transcription (Woychik et al., 1990; Zaros et al., 2007; Cuevas-Bermúdez et al., 2017; Martínez-Fernández et al., 2017). This must also account for plant NRPD/E5 subunits (RNA pol IV/V) with roles in gene silencing and RNA-directed DNA methylation, RdDM (Huang et al., 2009; Pikaard and Tucker, 2009; Lopez et al., 2011; Zhou and Law, 2015). Notably, subunit NRP6, one of the subunits common to RNA pols having a bacterial homolog (the ω subunit), is thought to be important for RNA pols assembly and stability and to play specific roles in transcription (Werner and Grohmann, 2011; Garrido-Godino et al., 2013; Nouraini et al., 1996; Lanzendorfer et al., 1997; Minakhin et al., 2001). It is worth noting that the Rpb8 common subunit is described as being eukaryote-specific, although an Rpb8 archaeal ortholog, called G or Rpo8, has been identified in Crenarchaeota, and it is thought to be a protein that appears at an early step in eukaryotic evolution (Koonin et al., 2007; Kwapisz et al., 2008).

The olive tree (*Olea europaea* L.) is one of the most important fruit trees in the Mediterranean Basin. Olive cultivation is important in economic, agronomic, and agro-ecological terms in many countries. Given its health benefits and economic impact in Mediterranean countries, olive oil is probably the most important vegetable oil in the world (Conde et al., 2008). Extra virgin olive oil is appreciated worldwide, thanks to its benefits for human health (Donaire et al., 2011). Its global demand continuously rises, and “Picual” is one of the most extensively cultivated olive varieties, thanks to the organoleptic properties of its extra virgin olive oil and excellent oxidative stability (Gutiérrez et al., 2001; Talhaoui et al., 2016). The cultivar “Picual,” of Andalusian origin, is the leading cultivar in Spain that accounts for 50% of national oil production and 20% of world oil production. The *O. europaea* genome is diploid, with 46 chromosomes (2n), whose size ranges from 1.48 to 2.2 Gb depending on the sequenced variety (Rugini et al., 1996; Loureiro et al., 2007). Recently, the “Picual” genome has been reported (Jiménez-Ruiz et al., 2020). “Picual” is a diploid organism (2n = 2x = 46) whose genome is larger than that of *O. europaea* var. *sylvestris*, with an estimated size of 1.68 Gb and 79667 genes, more than wild genomes (Jiménez-Ruiz et al., 2020). The cultivated olive genome results from two independent whole-genome duplications (WGDs) from around 62 and 25 million years ago, but with very recent partial genome duplications (Unver et al., 2017; Jiménez-Ruiz et al., 2020). “Picual” displays an excellent capacity to adapt to a wide variety of growth conditions, soils, stress, or pathogenic agent infections. “Picual” is sensitive to *Verticillium dahliae* infection, as are most olive cultivars, which has an important impact on economy or ecology. Adaptation to all these situations results from complex transcriptional responses, among other regulatory events (Leyva-Pérez et al., 2015; Jiménez-Ruiz et al., 2017; Jiménez-Ruiz et al., 2018).

Although extensive global transcriptomic studies have been performed on “Picual” (tissues and organs, abiotic cold stress, biotic stress by *V. dahliae* infection) and in early development from seeds, very little is known about transcriptional machinery regulation, and specifically about regulation of RNA pols. Taking advantage of this cultivar’s recently reported genome (Jiménez-Ruiz et al., 2020) and its economic, agronomic, and ecological importance, at least in the Mediterranean Basin, we analyzed the RNA pol gene composition and gene expression regulation in different relevant situations that impact growth or are of interest for genetic improvement. To do so, we focused on subunits common to RNA pols by understanding that they are shared by different RNA pols and that plants contain several specific paralogs of some RNA pol subunits. Based on both “Picual” genome and RNA-Seq datasets from tissues and organs, abiotic cold stress, biotic stress by *V. dahliae* infection, and early development transcriptomic studies, we investigated and described the existence of multigene families coding for subunits common to RNA pols and elucidated their differential transcriptional responses under these conditions.

## Materials and Methods

### Genome-Wide Identification of Genes and Proteins for Common Subunits of RNA Polymerases in the Olive “Picual” Genome

Arabidopsis RNA pol common subunit genes were identified in The Arabidopsis Information Resource (TAIR) (www.arabidopsis.org). Protein sequence queries were used to search for homologs by BlastP with an E value of <1 × 10^−5^ to identify common subunit proteins of plant RNA polymerases.

The common subunits of the *Arabidopsis thaliana* RNA pols used as queries were NRPA/D5, At3g22320; NRPE5, At3g57080; NRPE5-Like, At2g41340; NRPA/E6a, At5g51940; NRPA/E6b, At2g04630; NRPA/E8a, At1g54250; NRPA/E8b, At3g59600; NRPA/E10, At1g11475; NRPB10-like, At1g61700; NRPA/E12a, At5g41010; NRPB12-like, At1g53690. The identified sequences of the common subunits of Arabidopsis RNA pols were subsequently employed as queries to recover their homologs from the “Picual” genome using BlastP searches, available at the OliveTreeDB website (https://genomaolivar.dipujaen.es/db/index.php). Genomic, cDNA, CDS, and protein sequences were obtained for each common subunit of the RNA pols.

The retrieved “Picual” NRP5 sequences were aligned to other plant RNA pol sequences for further analyses. The other common subunits of plant RNA polymerases herein used were NRPB5a_*Zea mays*, NP_001141164; NRPB5b_*Zea mays*, NP_001132429.1; NRPE_*Zea mays*, ACG37268; NRPE5_*Pinus canariensis*, AJA90785.1; NRPE5_*Ginkgo biloba*, AJA90777.1; NRPE5_*Ephedra trifurca*, AJA90766.1; NRPE5_*Cycas revoluta*, AJA90761.1; NRP5A-like.a (*O. europapea sylvestris*), XP_022875925; NRP5A-like.c (*O. europapea sylvestris*), XP_022871082.1; NRPE5 (*O. europapea sylvestris*) XP_022872077.1.

### RNA-Seq Analysis

All RNA-Seq datasets used for the different studies have been previously described and are indicated later. The transcriptional steady-state levels of the olive cultivar “Picual” genes for subunit common to the RNA pols in organs/tissues were obtained from previously described datasets (Ramírez-Tejero et al., 2020). In brief, samples were collected from the roots, stems, meristems, leaves, flowers, and fruit of three healthy 10-year-old “Picual” olive trees under field conditions at the World Olive Germplasm Collection (WOGC) of the Andalusian Institute of Agricultural and Fisheries Research and Training (IFAPA), Córdoba, Spain. Two biological replicates (consisting of an equilibrated pool of three plant RNAs per sample) were sequenced.

The datasets described later were used for early development plant samples (Jiménez-Ruiz et al., 2018). For plant material preparation purposes, the seeds from the open pollinated cultivar Arbequina were induced to geminate at the Agrarian Research and Training Center (IFAPA) in Churriana, Spain. Seedlings were grown *in vitro* under chamber conditions with a 16-h photoperiod of fluorescent light at a constant temperature of 25°C until they were 2 months old. Then they were potted and grown in a conditioned greenhouse (25°C). The aerial parts of 10 plants were collected 1, 2, 3, 4, 5, and 6 months after seed activation. Two biological replicates of 10 pooled plants per sample were sequenced.

The *V. dahliae*–infected plants were obtained at the Department of Crop Protection, Institute for Sustainable Agriculture, Córdoba, Spain, and data from previously reported datasets were used (Jiménez-Ruiz et al., 2017). 40 plants were infected by root-dip inoculation in a conidial suspension (10^7^ conidia ml^−1^) of defoliating *V. dahliae* isolate V937I. As a control group, 40 non-inoculated plants were handled in the same way to be used in the absence of the pathogen cited before. For each biological replicate, roots from three plants were pooled from the control or after 2 and 7 days postinfection, and the cDNA from the samples was sequenced.

The cold stress and cold acclimation data were obtained from the previously described datasets (Leyva-Pérez et al., 2015) and were obtained as the previous *V. dahliae*–infected plants at the Department of Crop Protection, Institute for Sustainable Agriculture, Córdoba, Spain. For this purpose, thirty-five 4-month-old potted olive “Picual” cultivar plants were used, acclimated at 24°C, and then incubated with a 14-h photoperiod of fluorescent light at 65 μmol m^2^ s (10°C day/4°C night) for 10 days and constant 76–78% relative humidity. Another group of 15 plants was used as the control treatment. Aerial tissues were harvested at 0, 10, and 24 days, and three plants were pooled for each biological replicate for RNA extraction and sequencing purposes.

For RNA sequencing, samples were immediately frozen in liquid nitrogen and stored at −80°C. Total RNA was extracted with the Spectrum Plant Total RNA Kit (Sigma-Aldrich, St. Louis, MO, United States) according to the manufacturer’s instructions. Two technical replicates of each sample were sequenced by paired-end sequencing (101 × 2) in an Illumina^®^ HiSeq sequencer (Illumina, San Diego, CA, United States) at Sistemas Genómicos company (Valencia, Spain).

The expression analysis was performed with DNAstar (ArrayStar 17, Rockville, MD, United States) for the RNA-seq analyses (www.dnastar.com). Reads were mapped to the “Picual” genome as reference Oleur061 (Jiménez-Ruiz et al., 2020). Mapping was performed with high-stringency parameters to differentiate between highly similar paralogs, k-mer = 63 and 95% matches. Data were normalized using parameter reads per kilobase of transcript, per million mapped reads (RPKM). A basal expression level of log_2_ RPKM = −2 was considered. Therefore, the genes with expression values above this threshold level were considered expressed, whereas those with values that equaled or were below the threshold level were considered not expressed.

### Data Availability Statement

The RNAseq data are available at the National Center for Biotechnology Information (NCBI) Gene Expression Omnibus (GEO). The organs/tissues data are available with accession numbers GSE140648, GSM4176229, GSM4176230, GSM4176231, GSM4176232, GSM4176233, GSM4176234, GSM4176235, GSM4176236, GSM4176237, GSM4176238, GSM4176239, and GSM4176240 for Project PRJNA556567 (Ramírez-Tejero et al., 2020).

The early development data are available with accession Numbers (NCBI: SAMN07603885, SAMN07603886, SAMN07603887, SAMN07603888, SAMN07603889, SAMN07603890, SAMN07603891, SAMN07603892, SAMN07603893, SAMN07603894, SAMN07603895, and SAMN07603896) for Project PRJNA401310 (Jiménez-Ruiz et al., 2018). The data corresponding to the response to cold stress and to *V. dahliae* infection are available with accession numbers SRR1525051, SRR1525052, SRR1524949, SRR1524950, SRR1524951, SRR1524952, SRR1525086, SRR1525087, SRR1525113, SRR1525114) SRR1525231, SRR1525237, SRR1524947, SRR1524948, SRR1525213, SRR1525114, SRR1525224, SRR1525226, SRR1525284, SRR1525285, SRR1525286, SRR1525287, SRR1525415, SRR1525416, SRR1525436, and SRR1525437 (Leyva-Pérez et al., 2015; Jiménez-Ruiz et al., 2017).

### Phylogenetic Analysis

For phylogenetic analysis, the Phylogeny.fr interface was used (www.phylogeny.fr) (Dereeper et al., 2008). To do so, amino acid sequences were aligned with MUSCLE, and Gblocks was used for alignment curation. The phylogenetic tree was reconstructed using the maximum parsimony method with software PhyML (Guindon et al., 2010). Finally TreeDyn for tree drawing was used.

The amino acid sequences used were: Arabidopsis subunits common to RNA polymerases NRPA/D5, At3g22320, NRPE5, At3g57080, and NRPE5-Like, At2g41340, as well as NRPB5a _*Zea mays*, NP_001141164; NRPB5b _*Zea mays*, NP_001132429.1; NRPE_*Zea mays*, ACG37268; NRPE5_*Pinus canariensis*, AJA90785.1; NRPE5_*Ginkgo biloba*, AJA90777.1; NRPE5_*Ephedra trifurca*, AJA90766.1; NRPE5_*Cycas revoluta*, AJA90761.1; NRP5A-like.a (*Olea europapea sylvestris*), XP_022875925; NRP5A-like.c (*Olea europapea sylvestris*), XP_022871082.1; NRPE5 (*Olea europapea sylvestris*) XP_022872077.1.

## Results

### Identification of Genes for Common Subunits of RNA Polymerases

In order to identify the subunits common to all the RNA pols from olive (NRP5, NRP6, NRP8, NRP10, NRP12), we used the recently reported “Picual” olive genome (Jiménez-Ruiz et al., 2020). We searched for loci containing the ORFs putatively coding for subunits common to RNA pols using BLAST search and the corresponding subunits common to RNA pols from *A. thaliana* as queries.

**FIGURE 1:**
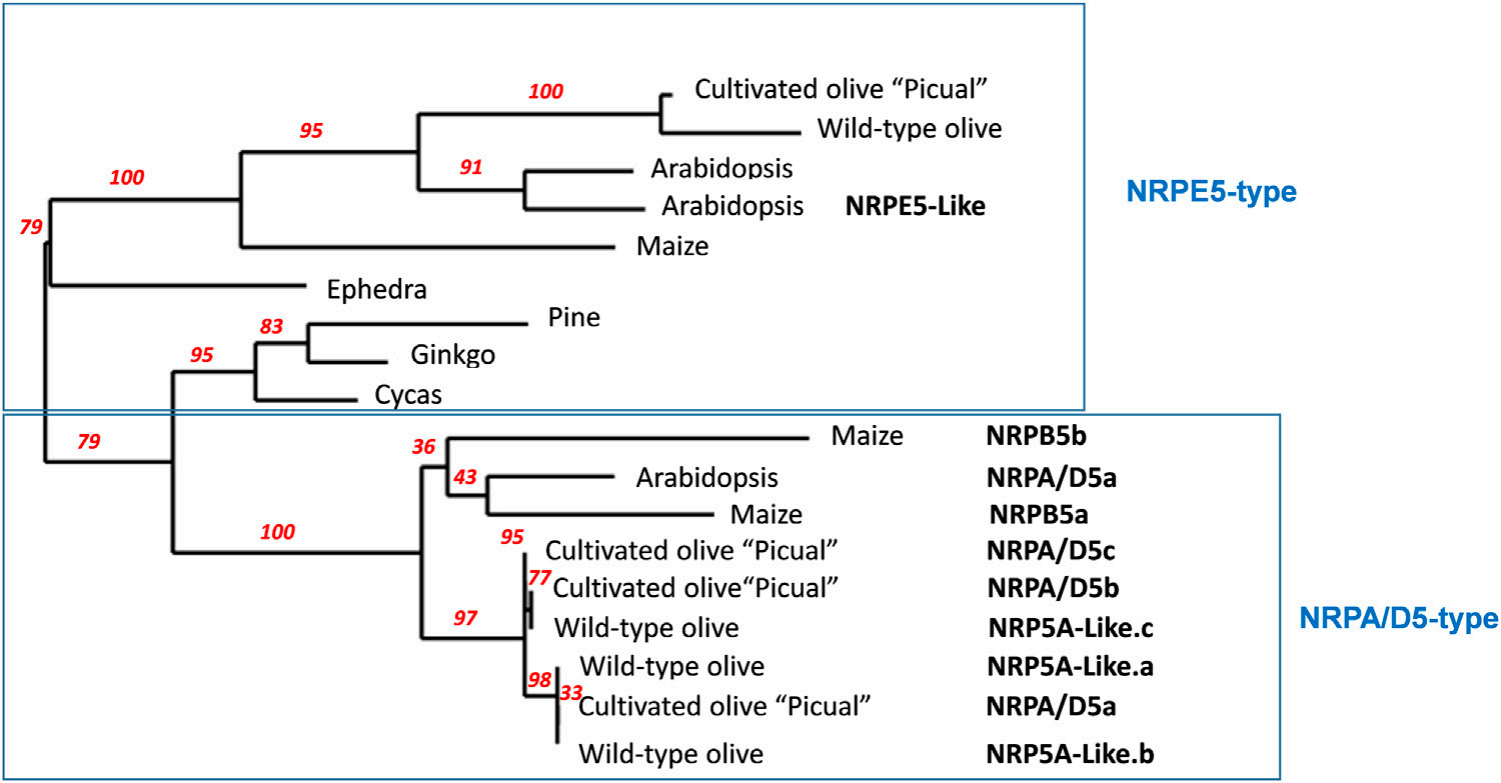
Schematic phylogenetic diagram of NRP5 genes. NRP5 sequences were aligned with MUSCLE, and the unrooted phylogenetic tree was reconstructed using the maximum likelihood method with the PhyML algorithm. The numbers at the nodes represent the percentage bootstrap values (only those higher than 50% were represented). The reliability for the internal branch was assessed using 100 bootstrap replicates. The NRPE5-type denoted the RNA pol V–specific subunit, including the corresponding NRPE5-like described in Arabidopsis. Cultivated olive corresponds to *Olea europaea sylvestris* (Unver et al., 2017). Arabidopsis RNA polymerase common subunits NRPA/D5, At3g22320, NRPE5, At3g57080 and NRPE5-Like, At2g41340 were used and NRPB5a _*Zea mays*, NP_001141164; NRPB5b _*Zea mays*, NP_001132429.1; NRPE_*Zea mays*, ACG37268; NRPE5_*Pinus canariensis*, AJA90785.1; NRPE5_*Ginkgo biloba*, AJA90777.1; NRPE5_*Ephedra trifurca*, AJA90766.1; NRPE5_*Cycas revoluta*, AJA90761.1; NRP5A-like.a (*Olea europapea sylvestris*), XP_022875925.1; NRP5A-like.b (*Olea europapea sylvestris*), XP_022875924.1; NRP5A-like.c (*Olea europapea sylvestris*), XP_022871082.1; NRPE5 (*Olea europapea sylvestris*) XP_022872077.1.

Arabidopsis has six genes that putatively encode NRP5 subunits (Larkin et al., 1999; Ream et al., 2009). However, only one subunit shared by RNA pols I-IV, NRPA/D5, and a second one specific to RNA pol V, NRPE5, have been identified in proteomic and functional analyses (Larkin et al., 1999; Ream et al., 2009; Law et al., 2011; Zhang et al., 2013). More recently, an NRPE5-like subunit was identified in proteomic analyses as being a component of RNA pol V, while this is not the case for two other putative NRPE-like subunits (Law et al., 2011). By using Arabidopsis NRPA/D5 as a query, we identified three putative genes coding for the “Picual” olive homolog subunits with identities falling within the 74–79% range. Concomitantly, we named them NRPA/D5a/b/c (Table 1 and Supplemental Figure S1). The NRPE5 homolog search permitted the identification of only one putative gene coding for the NRPE5 subunit with about 62% identity (Table 1 and Supplemental Figure S1). However, and unlike Arabidopsis, no NRPE5-like subunits were identified (58% and 68% identities between the Arabidopsis NRPE5-like and olive and the Arabidopsis NRPE5 subunits, respectively; Table 1 and Supplemental Figure S1). Furthermore, the existence of two classes of NRP5 subunits in olive was corroborated by the phylogenetic analysis (Figure 1). In addition, the NRPE5 subunit maintained the short N-terminal extension described for the plant NRPE5, as compared to NRPA/D5 (Supplemental Figure S2), which was suggested to be important for protein stability *in vivo* in Arabidopsis (Ream et al., 2009).

**TABLE 1:** Identified common subunits of RNA polymerase from olive.

Subunit		Gene accession	Protein size (amino acids)	mRNA expression			
				Organs and tissues	Biotic stress (*V. dahliae* infection)	Abiotic stress (Cold acclimation)	Development
NRP5	NRPA/D5a	Oleur061Scf3785g07006.1	203	+	+	+	+
	NRPA/D5b	Oleur061Scf0084g07007.1	217	+	+	+	+
	NRPA/D5c	Oleur061Scf3324g05023.1	206	+	+	+	+
	NRPE5	Oleur061Scf4420g01012.1	228	+	+	+	+
NRP6	NRPA/E6a	Oleur061Scf2238g07030.1	141	+	+	+	+
	NRPA/E6b	Oleur061Scf0173g01014.1	143	+	+	+	+
	NRPA/E6c	Oleur061Scf0677g02024.1	131	+	+	+	+
	NRPA/E6d	Oleur061Scf5121g00005.1	113	+	+	+	+
	NRPA/E6e	Oleur061Scf0350g03011.1	119	−	−	−	−
NRP8	NRPA/E8a	Oleur061Scf0592g02022.1	165	+	+	+	+
	NRPA/E8b	Oleur061Scf0022g02019.1	148	+	+	+	±
	NRPA/E8c	Oleur061Scf5855g00014.1	148	+	+	+	+
NRP10	NRPA/E10a	Oleur061Scf0656g01027.1	71	+	+	+	+
	NRPA/E10b	Oleur061Scf3000g03019.1	63	+	+	+	+
NRP12	NRPA/E12a	Oleur061Scf2481g09006.1	127	+	+	+	+
	NRPA/E12b	Oleur061Scf2607g00039.1	97	+	+	+	+
	NRPA/E12c	Oleur061Scf1394g00001.1	72	±	+	±	±
	NRPA/E12d	Oleur061Scf1987g03032.1	94	−	−	−	−

+ Means expressed in all samples. ± Means expressed in some samples. – Means not expressed in any sample.

Based on Blast analysis using *A. thaliana* common subunits of RNA polymerases as queries: **NRPA/D5,** At3g22320**; NRPE5,** At3g57080**; NRPE5-Like,** At2g41340**; NRPA/E6a,** At5g51940 **; NRPA/E6b,** At2g04630; **NRPA/E8a**, At1g54250; **NRPA/E8b**, At3g59600; **NRPA/E10**, At1g11475; **NRPB10-like**, At1g61700 ; **NRPA/E12a**, At5g41010 ; **NRPB12-like**, At1g53690.

Two NRP6 subunits, NRPA/E6a and NRPA/E6b, have been described in Arabidopsis (Ream et al., 2009; Law et al., 2011; Ream et al., 2015). By using them as queries, we identified five putative NRP6 coding genes in olive (Table 1 and Supplemental Figure S1). Three of the corresponding subunits (named NRPA/E6a/b/c) showed high identity among them (88–96%) and ranged from 69 to 79% identity in relation to the Arabidopsis NRPE6 subunits. Strikingly, the other two subunits (named NRPA/E6d/e) were small in size, with 113 and 119 amino acids. NRPA/E6d showed about 59% identity compared to the Arabidopsis subunits, while NRPA/E6e displayed the least identity of about 49%.

Two NRP8 subunits in Arabidopsis have been shown to form part of all five RNA pols: NRPA/E8a and NRPA/E8b (Ream et al., 2009; Law et al., 2011; Ream et al., 2015). In olive, three putative coding genes for the NRPA/E8a/b/c subunits were identified. The NRPA/E8a identity range was 53–55% with the Arabidopsis subunits, while NRPA/E8b/c range was 45% (Table 1 and Supplemental Figure S1).

In Arabidopsis, two NRP10 subunits (NRPA/E10 and NRPB10-like) and two NRP12 subunits (NRPA/E12 and NRPB12-like) have been detected (Ream et al., 2009; Law et al., 2011; Ream et al., 2015), although the association of NRPB10-like and NRPB12-like with RNA pols remains controversial (Ream et al., 2015). By using them as queries, two putative coding genes for NRPA/E10a/b have been identified in the olive “Picual” with identities within the 90–95% range with their Arabidopsis homologs (Table 1 and Supplemental Figure S1). Conversely, four putative coding genes for the NRPA/E12a/b/c/d subunits were found in “Picual,” whose identities were greater with Arabidopsis NRPA/E12 (67–90%) than with NRPB12-like (52–71%) (Table 1 and Supplemental Figure S1). Interestingly, NRPA/E12d showed the fewest identities with the other NRPA/E12 olive subunits when they were all compared.

### The Genes for Subunits Shared by RNA Polymerases are Spatially and Temporally Regulated

In line with the aforementioned data, we have speculated that several members of each distinct subunit common to RNA pols existed in the olive cultivar. To explore whether the corresponding putative coding genes were functional and expressed, we analyzed their spatial and temporal expression patterns.

We first investigated the expression of genes putatively coding for the different NRP5, NRP6, NRP8, NRP10, and NRP12 subunits from the “Picual” olive cultivar by analyzing their mRNA levels in several organs (fruits, flowers, leaves, roots, stems) and tissues (meristems) with the RNA-Seq data from a previously detailed transcriptomic analysis (Ramírez-Tejero et al., 2020).

**FIGURE 2:**
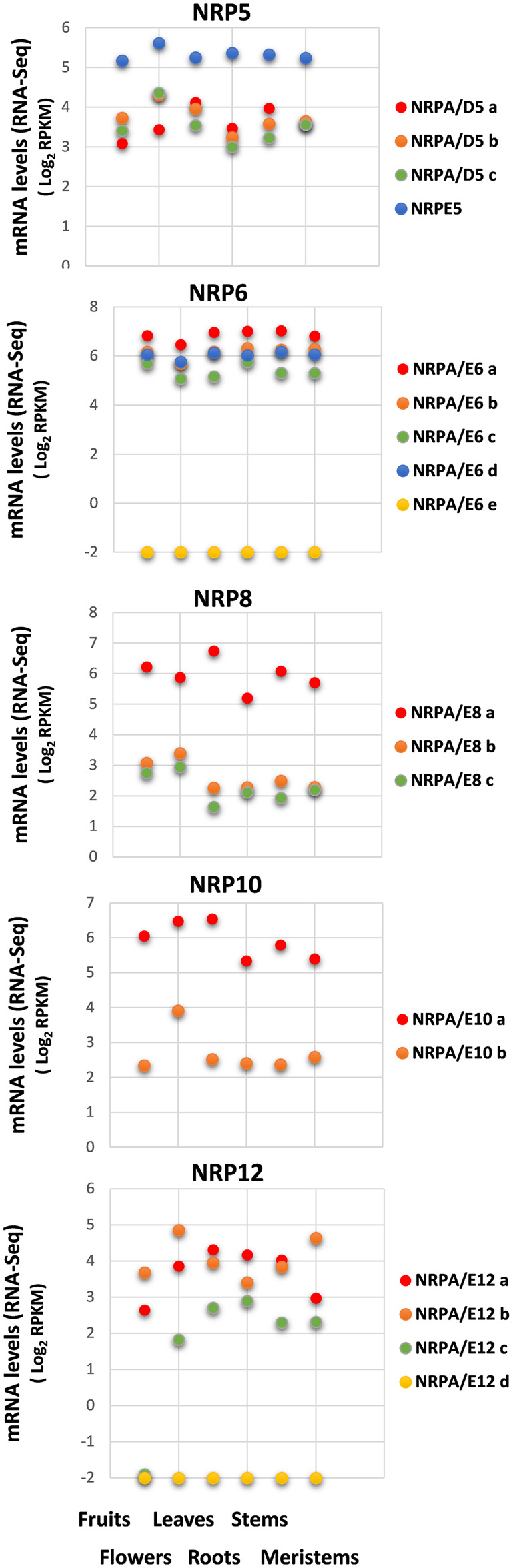
Gene expression pattern of subunits common to RNA polymerases in different organs and tissues. Data from previously published RNA-Seq datasets (Ramírez-Tejero et al., 2020). Expression profile of the different NRP common subunit genes from olive cultivar “Picual” in fruit, flowers, leaves, roots, stems, and meristems. Data correspond to reads per kilobase of transcript per million mapped reads (RPKM). Data are represented as log_2_ (RPKM). Values ≤ −2 are considered no expression and are represented by a value of −2.

As shown in Figure 2, all the identified NRP genes from olive were expressed in all the analyzed organs and tissues, except Oleur061Scf0350g03011.1 for NRPA/E6e and Oleur061Scf 1987g03032.1 for NRPA/E12d, which were not expressed for either condition. A complex expression pattern was also observed when we compared the genes of the different NRP subunits. However, when independently comparing each subset of NRP genes corresponding to each NRP subunit, our results showed that the different paralog genes tended to maintain similar expression levels in the different organs and tissues, except for the NRP12 genes and NRPA/E10b.

Regarding expression levels, NRPE5 showed the highest mRNA levels of all the NRP5 genes, while the other three NRPA/D5 genes were similarly expressed in the different organs and tissues. Moreover, the gene for the NRPA/E8a subunit was more highly expressed than the other two identified NRPA/E8 genes. This was also the case for the NRPA/E10a gene *versus* NRPA/E10b. Notably, no major differences in expression levels were observed for the four expressed NRP6 coding genes. Finally, the NRP12 genes presented the biggest differences when comparing their expression levels in the different analyzed organs and tissues. The NRPA/E12c gene was not expressed in fruits and was similarly expressed in the other analyzed organs and tissues. These differences in expression levels did not seem to maintain a relation with the total mRNA amount detected in any analyzed organ and tissue (Supplemental Figure S3).

These results collectively suggested that most NRP coding genes were expressed, with no major differences between different organs and tissues for each gene, except the NRP12c gene that was not expressed in fruits, and NRPA/E10b that was overexpressed in flowers. On the contrary, a clear spatial regulation with evident differences in the expression levels between paralog genes was observed, thus implying that some of these subunits have major contribution. We further investigated whether NRP common subunit genes from the olive cultivar could be temporally regulated by exploring their expression patterns using previously reported and corroborated datasets during the early juvenile development period from 1-month to 6-month seedlings (from germinated embryos to juvenile trees), the end of which corresponds to the juvenile development stage (Jiménez-Ruiz et al., 2018). Note that a complete transcriptomic study of olive development during the early juvenile period demonstrated that after 3–4 months of development, all plant structure and cell and organ differentiation have occurred, and thus, the juvenile tree development from seed is complete (Jiménez-Ruiz et al., 2018).

**FIGURE 3:**
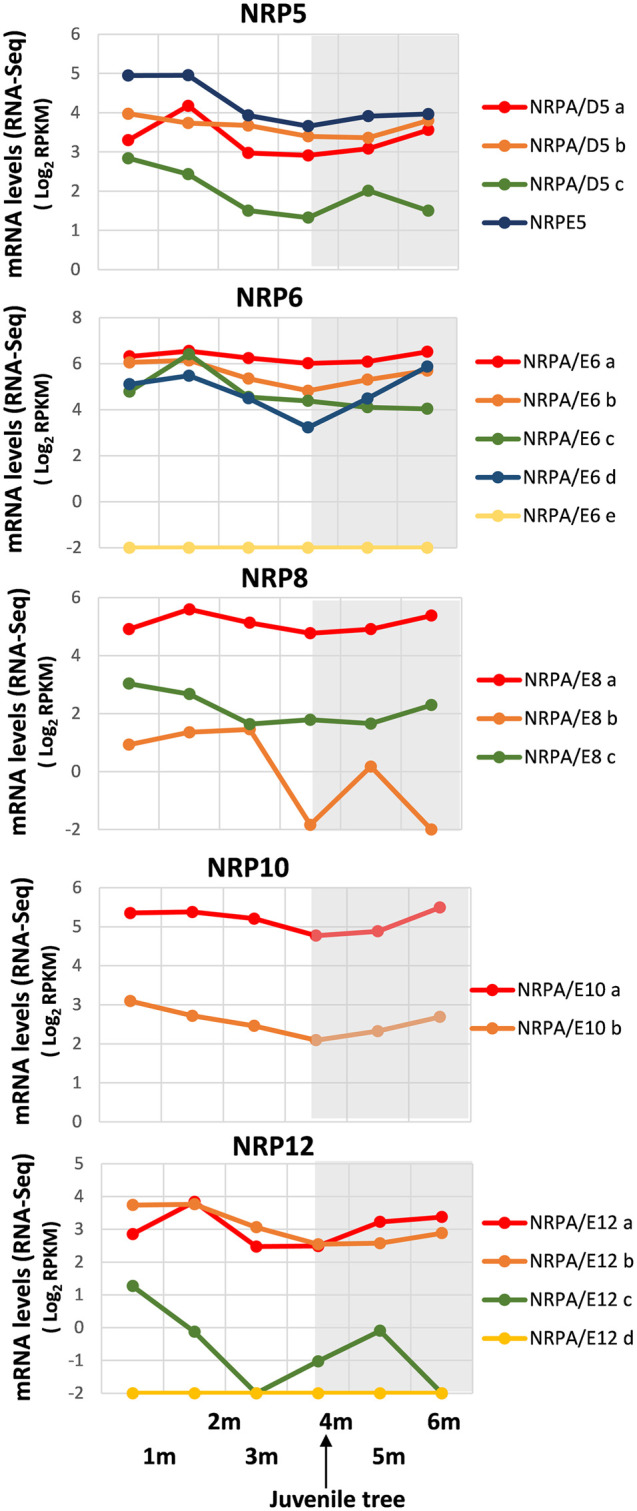
Gene expression pattern of subunits common to RNA polymerases during early development from germinated embryos to juvenile trees. Data from the RNA-Seq datasets (Jiménez-Ruiz et al., 2018). Expression profile of the different NRP genes from olive cultivar “Picual” during early development 1, 2, 3, 4, 5, and 6 months after seed activation. Data correspond to reads per kilobase of transcript per million mapped reads (RPKM). 4 months corresponds to the time considered for plant to be a juvenile tree. Data are represented as log_2_ (RPKM). Values ≤ −2 are considered no expression and represented by a value of −2. The gray square represents the late developmental period once plants were juvenile trees.

As shown in Figure 3, a general decrease in NRP gene expression was observed until development at 4 months, although differences were found in mRNA levels among distinct gene paralogs. We were unable to exclude some gene expressions not being mainly altered, as with NRPA/E6a. It is worth noting that 4 months corresponded to completed juvenile tree development from seed (Jiménez-Ruiz et al., 2018). Interestingly, some differences were evident: NRPA/D5a, NRPA/D6c, and NRPA/E12a gene expressions peaked at 2-month development and then lowered, while NRPA/E8b and NRPA/E12c expressions drastically decreased. These results could account for major transcriptional activity during early development from seed before later reaching the levels maintained mainly in juvenile and/or adult trees.

Notably, after juvenile tree formation (between 4 and 6 development months), the expression of most NRP genes increased or remained unaltered, except for NRPA/E6c, whose slow decrease in gene expression continued from 2 development months. In addition, the NRPA/D5c expression pattern differed by lowering between 5 and 6 development months. This also occurred for NRPA/E8b and NRPA/E12c, although their expression drastically dropped, or even disappeared, after 3 and 1 development months, respectively, before increasing to 5 development months.

Furthermore, we corroborated that genes NRPA/E6e and NRPA/E12c were not expressed during development from germinated embryos to juvenile trees, and NRPE5, NRPA/E8a, NRPA/E10a, and NRPA/E12a and b were the most expressed gene paralogs, as observed under other conditions analyzed herein. Altogether, these data demonstrated that most NRP common subunit genes from olive were expressed and were spatially and temporally regulated.

### The Genes for Subunits Shared by RNA Polymerases are Regulated by Stress Conditions

Biotic and abiotic stresses impact olive tree cultivars, leading to vast economic loss and agronomic damage (López-Escudero and Mercado-Blanco, 2011; Trapero et al., 2013). Accordingly, we investigated whether NRP common subunit genes were expressed under biotic and abiotic stresses.

A wide variety of biotic constraints affects olive cultivation, including *Verticillium* wilt of olive caused by the pathogenic fungus *V. dahliae*, which is detected in almost all the regions where olive culture exists, and is one of the most harmful diseases that affect this woody crop, leading to vast economic loss and agronomic damage, particularly in the Mediterranean Basin (López-Escudero and Mercado-Blanco, 2011). Most olive tree cultivars are susceptible to this disease, including “Picual” (Trapero et al., 2013).

**FIGURE 4:**
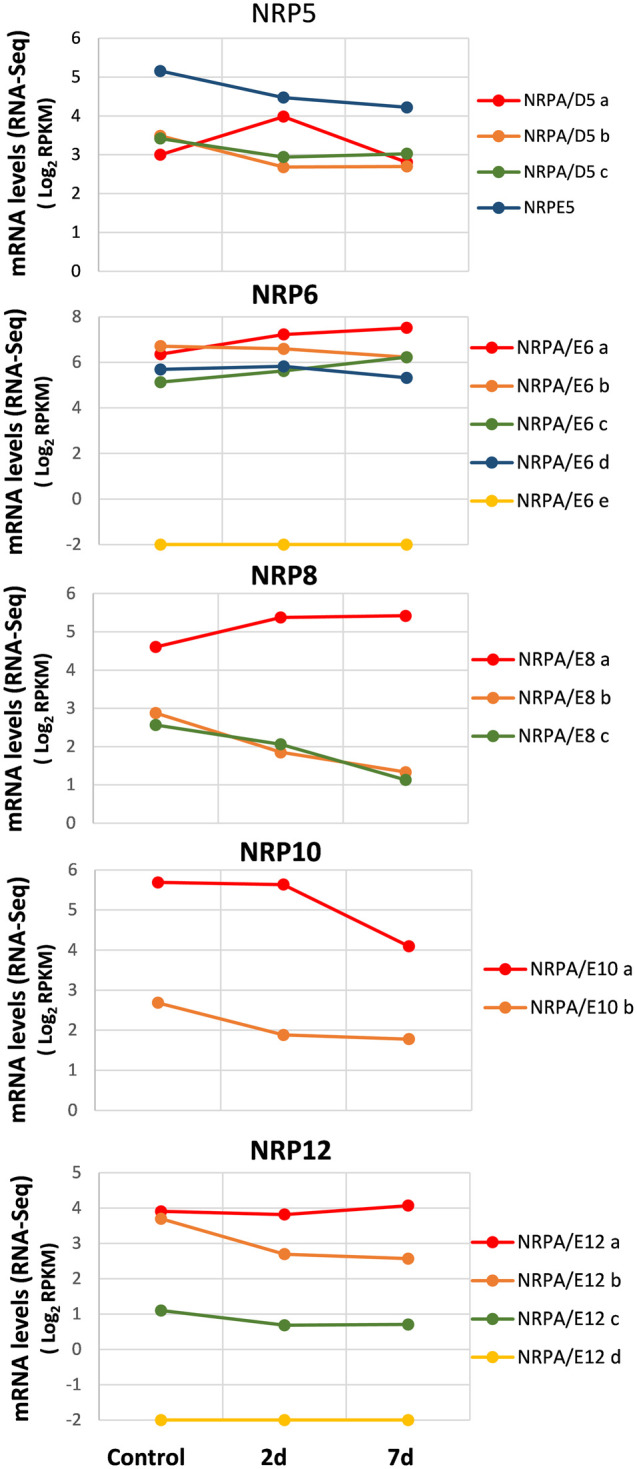
Gene expression pattern of subunits common to RNA polymerases during the *V. dahliae* early infection process. Data from previously published RNA-Seq datasets (Jiménez-Ruiz et al., 2017). Expression profile of the different NRP genes from olive cultivar “Picual” 1 and 7 days after *V. dahliae* infection. The control corresponds to the control group of non-inoculated plants, handled in the same way as in the absence of the pathogen. Data correspond to reads per kilobase of transcript per million mapped reads (RPKM). Data are represented as Log_2_(RPKM). Values ≤ −2 are considered no expression and represented by a value of −2.

We collected data from recently published genome-wide transcriptomic studies conducted during infection and the plant–*V. dahliae* interaction (Jiménez-Ruiz et al., 2017; Leyva-Pérez et al., 2018), we investigated the expression pattern of genes for subunits shared by RNA polymerases from olive cultivars associated with biotic stress during *V. dahliae* root infection. We also used the RNA-Seq data from the total RNA extracted from the roots of three groups of three randomly selected plants after 48 h and 7 days of infection, as well as from the control plants, taken as time 0 before infection (Jiménez-Ruiz et al., 2017). As shown in Figure 4, the analysis of the control plants (time 0 before infection) corroborated our previous results observed in roots (Figure 2). Notably, in the susceptible “Picual” cultivar, the gene expression for most NRP subunits decreased during infection and plant–*V. dahliae* interaction (Figure 4). On the contrary, in the resistant cultivar “Frantoio,” none of the NRP common subunit genes decreased their expression during *V. dahliae* infection, suggesting that this differential expression may contribute to the resistance or sensitivity to *V. dahliae* infection (Supplemental Figure S4; compare with Figure 4). However, some exceptions were observed in the “Picual” NRP expression pattern (Figure 4). This was the case of the NRPA/D5a gene, which was upregulated 2 days after infection, and the levels lowered to those noted in the control (time 0) at seven days. The expression of genes NRPA/E6a, NRPA/E6c, and NRPA/E8a increased during infection, which suggests a specific response to *V. dahliae* infection in olive cultivars. Furthermore, NRPA/E12a, which was the most expressed of the four NRPA/E12 genes, showed no significant wide variation in gene expression during *V. dahliae* infection. This behavior differed from that of the other three NRP12 genes, whose expression decreased or was absent (NRPA/E12d). Notably, as observed before for the different organs and tissues, NRPE5, NRPA/E8a, and NRPA/E10a were still the most expressed genes among their paralogs, while NRPA/E6e was not expressed at all, which was also the case for the NRPA/E12d gene, as indicated before.

These results demonstrated the expression of most NRP common subunit genes during biotic stress by *V. dahliae* infection and suggest some NRPs’ major contribution to this response.

To further investigate the expression of NRP common subunit genes under abiotic stress, we have paid special attention to the olive cultivar response to abiotic cold stress as olive is sensitive to winter chilling temperatures, with severe leaf damage occurring at −7°C (D’Angeli et al., 2003). In this situation, an adaptive response, called cold acclimation (Chinnusamy et al., 2006), has evolved to overcome damage related to this abiotic stress.

In order to explore the expression pattern of genes for common subunits of RNA polymerases from the olive cultivar in response to abiotic cold stress, we used the RNA-Seq data from the whole-transcriptome analysis of cold acclimation in “Picual” plant leaves (Leyva-Pérez et al., 2015). To do so, the acclimated plants were subjected to cold stress, and aerial tissues (leaves) were harvested at 0 h (control), 24 h, and 10 days after cold stress (Leyva-Pérez et al., 2015). It is worth noting that cold stress symptoms were detected after 24 h of treatment, and plants completely recovered after 5 days (Leyva-Pérez et al., 2015).

**FIGURE 5:**
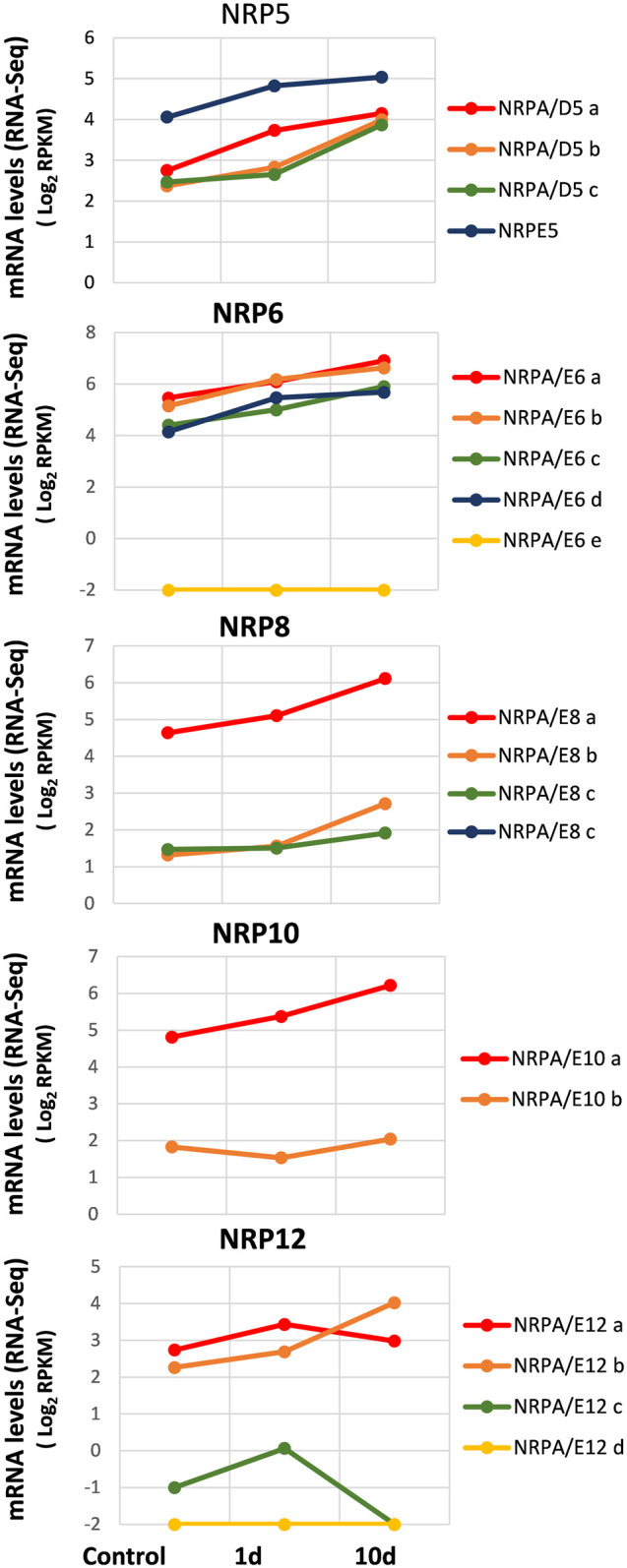
Gene expression pattern of subunits common to RNA polymerases under abiotic stress during cold acclimation. Data from previously published RNA-Seq datasets (Leyva-Pérez et al., 2015). Expression profile of the different NRP genes from olive cultivar “Picual” 1 and 10 days after cold acclimation. Control corresponds to the control group of acclimated plants at time 0. Data correspond to reads per kilobase of transcript per million mapped reads (RPKM). Data are represented as log_2_ (RPKM). Values ≤ −2 are considered no expression and represented by a value of −2.

As observed in Figure 5, our results at time 0 (control) corroborated mainly the aforementioned data in leaves (Figure 2). Furthermore, as observed in previously mentioned analyses, some NRP genes appeared to be the most expressed of their paralogs: NRPE5, NRPA/E8a, NRPA/E10a, and NRPA/E12a and b. In addition, as in previous analyses, NRPA/E6e and NRPA/E12d were not expressed. It is worth noting that these two features were also observed for all conditions analyzed herein. Interestingly, most expressed NRP genes showed a similar general response and were induced during cold acclimation (Figure 5). On the contrary, NRPA/E10b gene expression suggested not responding to this abiotic stress. This feature did not seem to be the result of NRPA/E10b gene constitutive expression because this gene responded to *V. dahliae* infection (Figure 4).

The NRP12 gene paralogs showed the most complex differentiated expression pattern (Figure 5), similar to that observed during *V. dahliae* infection (Figure 4). As shown, while NRPA/E12b gene expression was induced by cold acclimation, NRPA/E12a gene expression did not significantly alter. Strikingly, NRPA/E12c gene expression disappeared after 10 cold acclimation days.

Our results indicated a major global response to cold acclimation that resulted in NRP gene expression increasing mainly with time. Globally, our results demonstrated a major global transcriptional regulatory response to biotic and abiotic stresses.

## Discussion

Plants present a high genomic plasticity, and many species are polyploids or have been polyploids during some evolutionary events, such as olive trees. This fact is why unique genes in other eukaryote groups frequently have some paralog genes in plants. Duplicated genes may evolve and be silenced, or be specialized in a specific condition. Plants have two additional RNA pols (IV and V) to the general three-eukaryote ones as specialized enzymes that have evolved from RNA pol II. All eukaryotic RNA pols share five common subunits, which are mostly coded by unique genes. However, this is not true for plants, containing several paralog genes for these NRPs. Based on the marked agronomic, economic, and ecological interest of olive trees, we searched for genes of subunits shared by RNA polymerases (RNA pols are major elements in gene expression regulation) and studied if the different genes coding for each subunit were regulated and differentially expressed. For this reason and based on the recently reported cultivar “Picual” genome (Jiménez-Ruiz et al., 2020), we analyzed the composition of genes for common subunits of RNA polymerases and their expression patterns in several situations of interest, such as early development, organ/tissue profile, and biotic or abiotic stresses. Furthermore, globally analyzing their expression can help elucidate not only their contribution but also that of RNA pols to global transcriptional responses of interest to the cultivar “Picual.”

We identified distinct genes for all five subunits shared by RNA pols (Table 1). These results fall in line with those previously described for Arabidopsis and maize, or other angiosperms and gymnosperm plants (Haag et al., 2014; Wang and Ma, 2015; Ream et al., 2009), which suggests high divergence and large differences in evolutionary gene patterns for these different gene subunits (Wang and Ma, 2015). This feature must apply not only to RNA pols common subunits but also to any RNA pol subunits (Ream et al., 2009; Tucker et al., 2010; Ream et al., 2013; Haag et al., 2014; Ream et al., 2015; Wang and Ma, 2015). However, except for subunits five and ten, “Picual” possesses more genes than other plants for subunits 6 (five), 8 (four), and 12 (four) (Ream et al., 2009; Haag et al., 2014; Wang and Ma, 2015). These results agree with the olive cultivar genome resulting from two independent whole-genome duplication (WGD) events during domestication dating back some 62 and 25 million years ago, in addition to very recent partial genome duplications (Unver et al., 2017; Jiménez-Ruiz et al., 2020). On the contrary, angiosperms like Arabidopsis and maize genomes have resulted from one WGD and partial genome duplications (Jiao et al., 2011; Wang and Ma, 2015; Jiao, 2018; Ren et al., 2018). Only two genes for subunit ten were found in “Picual.” This suggest gene loss during evolution after gene duplications, which is described to be a general evolutionary mechanism (Wang and Ma, 2015; Julca et al., 2018). In angiosperms, subunit NRP5 gene duplication seemed to lead to the appearance of a gene coding for subunits NRPA/C5 and/or NRPA/D5 (RNA pols I-III or I-IV, respectively) and a second one for the NRPD/E5 subunit (RNA pols IV/V), whose duplication gave rise to the specific gene for the NRPE5 subunit (RNA pols V) in Arabidopsis (Wang and Ma, 2015). Similarly, in cauliflower, a specific NRPB5b subunit has evolved from an RNA pol II precursor into a functionally different subunit in RNA pol V (Huang et al., 2009). Notably, these occurrences are essential features for RNA pol IV and V specialization from RNA pol II (Tucker et al., 2010; Haag et al., 2014; Huang et al., 2015). The olive cultivar “Picual” only contains one gene that putatively codes for the NRPE5 subunit, according to the amino acid identity with Arabidopsis NRP5 proteins (Table 1 and Supplemental Figure S1), while three genes putatively coding for almost identical subunits (89–95%) showed a closer identity to the NRPA/D5 subunit. Accordingly, these data agree with a “Picual” NRPD/E5 coding gene being duplicated during the ancient WGD in gymnosperms (Jiao et al., 2011; Wang and Ma, 2015; Jiao, 2018; Ren et al., 2018), with the loss of at least one resulting duplicated gene after the second WGD occurring during *Olea*–*Fraxinus* ancestor speciation (Unver et al., 2017; Jiménez-Ruiz et al., 2020). Furthermore, this ancient WGD has been hypothesized to come from an ancestral allotetraploid produced by the hybridization of an ancestral *Fontanesia*-related species and an ancestral *Jasminum*–*Forsythia* species (Taylor, 1945; Unver et al., 2017; Jiménez-Ruiz et al., 2020). Conversely, the three “Picual” NRPA/D5 copies account for the very recent partial genome duplication, which may have occurred during domestication.

WGDs seem to account for the reciprocal loss or subfunctionalization of duplicated genes in different species, which enhances the adaptation of organisms to environmental challenge (Hegarty and Hiscock, 2008; Julca et al., 2018; Ren et al., 2018; Qiao et al., 2019). Gene inactivation mechanisms could occur for the “Picual” genes that putatively code for NRPA/E6e and NRPA/E12d as we were unable to detect mRNA expression under any analyzed condition (different organs and tissues, biotic and abiotic stress, plant development). Furthermore, we cannot rule out gene loss for other RNA pol subunits in “Picual” during the domestication period.

Except for the putative coding genes for NRPA/E6e and NRPA/E12d that are indicated earlier, all the other identified genes for the common subunits of RNA pols in “Picual” were expressed, according to our analyses of the different RNA-Seq datasets (Leyva-Pérez et al., 2015; Jiménez-Ruiz et al., 2017; Jiménez-Ruiz et al., 2018; Ramírez-Tejero et al., 2020). However, we cannot rule out that NRPA/E6e and NRPA/E12d can be expressed under other conditions, although it seems unlikely. Nevertheless, other genes for some Arabidopsis or maize RNA pol subunits have been found to be expressed according to mRNA analyses, but no corresponding proteins have been identified in proteomic and/or biochemical studies (Wang and Ma, 2015).

A holistic expression analysis reveals some interesting findings: *1*) most genes for subunits shared by RNA polymerases show similar expression patterns for most analyzed conditions, which suggests coordinated responses; *2*) global differences in the gene expression levels between the distinct paralogs are chiefly maintained under any of the analyzed conditions, albeit with specific differences for some common subunits of RNA polymerases, which indicate specific expression regulation for those NRP genes; *3*) one gene or two per subunit show the highest expression for any analyzed condition, save the NRP6 paralog genes with similar expression levels. This finding implies that major contribution to general gene expression depends on some gene paralogs; *4*) the NRP common subunit genes show spatial and temporal transcriptional regulation and respond to biotic and abiotic stress. Furthermore, certain specificities exist for each analyzed condition.

In terms of the spatial transcriptional regulation, gene expression of NRP common subunits was variable for different organs and tissues (Figure 2), and not only for different subunit genes, but also among diverse paralogs of the same NRP common subunit genes. Similarly, differences in NRP subunit expression in organs and tissues have also been observed for Arabidopsis, maize, and other plants (Ream et al., 2009; Ream et al., 2015; Wang and Ma, 2015). These results suggest that specific expression regulation for those NRP genes may be physiologically relevant in different organs and tissues and similarly in other growth conditions (*see* below).

Given cultivated olive trees’ agronomic importance, knowledge of early tree development gene regulation steps is relevant to manipulate and shorten the unproductive juvenile period (Moreno-Alías et al., 2010). Notably, transcriptomic analyses of seedlings during early development show a major alteration of gene expression in the first 3–4 months, and gene expression subsequently grows more stable once juvenile tree development from seed is complete (Jiménez-Ruiz et al., 2018). This also seems to be the case for most genes of subunits shared by RNA polymerases, with a slight general trend for a decrease in gene expression decrease during the 1- to 4-month periods (from seed to juvenile tree) and mostly maintained later. These results could imply greater transcriptional activity during early development from seed to juvenile tree being completed (4–6 months development), after which transcriptional activity lowered and remained the same in juvenile and/or adult trees. However, differences were observed for some genes (Figure 3). Our results also suggest a minor contribution of NRPA/E8b and NRPA/E12c to seedling development, which was mostly constrained to very early development steps as they were underexpressed 2 or 3 months after inducing germination. A specific transcriptional response in early development has been described for the genes involved in DNA methylation, which were upregulated during the 6-month follow-up (Jiménez-Ruiz et al., 2018). Interestingly, the role of RNA pols IV and V in RNA-directed DNA methylation has been clearly demonstrated (Huang et al., 2009; Ream et al., 2009; Haag and Pikaard, 2011; Haag et al., 2014; Zhou and Law, 2015) by acting during development (Pikaard and Tucker, 2009; Moo et al., 2012; Haag et al., 2014). However, the NRPE5 gene for RNA pol V (and/or IV) did not specifically and differently modulate its expression. This suggests that RNA pol V transcriptional regulation did not make any major contribution to olive development or that RNA pols are globally regulated at activity levels by the protein–protein interactions of transcriptional complexes, or even by posttranslational modification, rather than by the gene expression of RNA pol subunits.

Susceptible olive cultivar “Picual” responds to biotic stress provoked by *V. dahliae* infection by initiating a specific transcriptional stress response, similarly to that observed in other plants (Jiménez-Ruiz et al., 2017; Yuan et al., 2020). This complex transcriptional response may involve the regulation of some or all transcriptional machineries. Along this line, most NRP genes transcriptionally respond to this biotic stress as some decrease their expression in the plant–fungi interaction, while others specifically and distinctly respond by showing rapid upregulation at 2-days postinfection, or even remain generally unaltered (Figure 4). Notably, plants acquire immunity to pathogen infections, a response that involves the participation of different transcription factors and, at least, RNA pol V (Lopez et al., 2011; Amorim et al., 2017) *via* RdDM mechanism (Huang et al., 2009; Ream et al., 2009; Haag and Pikaard, 2011; Haag et al., 2014; Zhou and Law, 2015). However, subunit gene NRPE5, specific to RNA pol V (and/or IV), did not respond by specifically modulating its expression after *V. dahliae* infection in relation to other NRP5 genes (Figure 4) as could be expected. This result poses several considerations, as discussed above, during olive development. First is the notion that RNA pols are regulated at activity levels, although this did not seem to be a general feature of gene regulation due the differential transcriptional response of the NRP genes (Figure 4). Another possibility of NRPE5 expression not being specifically altered may suggest that RNA pol V transcriptional regulation did not make any major contribution to “Picual” infection by *V. dahliae* to mediate plant immunity, or could be the consequence of this olive tree being sensitive to *V. dahliae* (Leyva-Pérez et al., 2018)*.* In line with this, some genes implicated in *V. dahliae* infection have been seen to clearly upregulate in olive cultivar “Frantoio” which resists this fungus, while their expression remains unaltered or even decreases in “Picual” (Leyva-Pérez et al., 2018). Notably, none of the “Frantoio” NRP common subunit genes decreased their expression during *V. dahliae* infection, in contrast to the transcriptional response observed for “Picual,” suggesting that this differential expression may contribute to the resistance or sensitivity to *V. dahliae* infection (Supplemental Figure S4; compare with Figure 4).

The NRP genes for common subunits respond to abiotic cold stress (Figure 5) in line with general transcriptomic responses of this cultivar to cold (Leyva-Pérez et al., 2015) and with that of many other plants (Matsui et al., 2008; Qi and Zhang, 2019). Olive trees achieve a cold acclimation response that provokes metabolic, physiological, and developmental changes that are genetically controlled (Chinnusamy et al., 2006). In olive leaves, cold acclimation leads to a rapid cold stress response during the first 24-h exposure and a long-term expression response during 10-day cold exposure (Leyva-Pérez et al., 2015). However, while a general gene expression downregulation tendency is observed, the expression of most NRP common subunit genes increased during cold acclimation, with only clear distinct responses occurring for some NRP12 paralogs (Figure 5). These results suggest increased transcriptional activity that allowed olive plants to acclimatize and physiologically recover after initial cold stress exposure. We could speculate about this response being accompanied by increased cell cycle progression after cell cycle arrest by cold stress (Qi and Zhang, 2019), although the gene expression analyses performed with “Picual” during cold acclimation have revealed the downregulation of some cell cycle genes (Leyva-Pérez et al., 2015), and we found no alteration or minor increase in some CDK gene expression (not shown). It is worth noting that CDKs have been suggested to be relevant, at least *via* posttranscriptional modulation, during biotic and abiotic stress in plants (Kitsios and Doonan, 2011).

Although our data suggest the scenario of genes for subunits shared by RNA polymerases showing coordinated regulation to mediate the global transcriptional responses observed under different growth conditions or abiotic and biotic stress (Leyva-Pérez et al., 2015; Jiménez-Ruiz et al., 2017; Jiménez-Ruiz et al., 2018; Ramírez-Tejero et al., 2020), we found specific responses of some NRP common subunit paralog genes. These data suggest the contribution of some NRP common subunit genes to the transcriptional regulation mediated by RNA pols for olive “Picual” biology to adapt to different growth situations. Finally, based on our data, we cannot rule out that some NRP common subunit genes code for subunits with RNA pol specificity, which will be the goal of future studies.

## Data Availability

The datasets presented in this study can be found in online repositories. The names of the repository/repositories and accession number(s) can be found in the article/Supplementary Material.
