# Supplementary material for: Several Isoforms for Each Subunit Shared by RNA Polymerases are Differentially Expressed in the Cultivated Olive Tree (Olea europaea L.)
Source: Front Mol Biosci. 2021 Dec 20;8:679292. doi: 10.3389/fmolb.2021.679292 (PMC8721170; doi:10.3389/fmolb.2021.679292)
Supplement: Supplementary file 1 [file DataSheet1.docx]

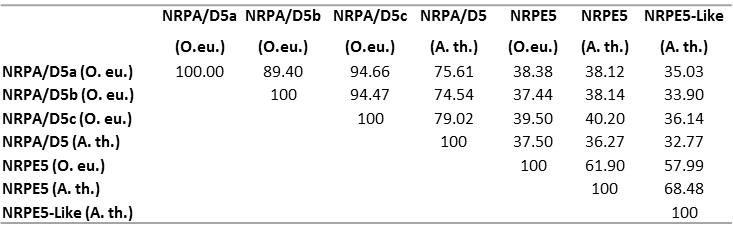

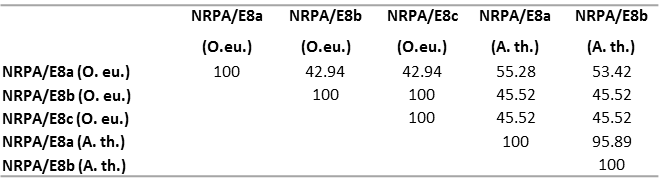

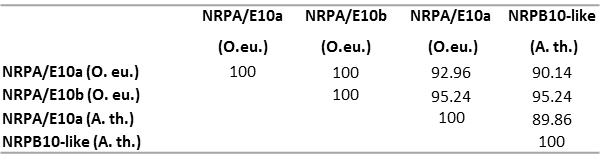


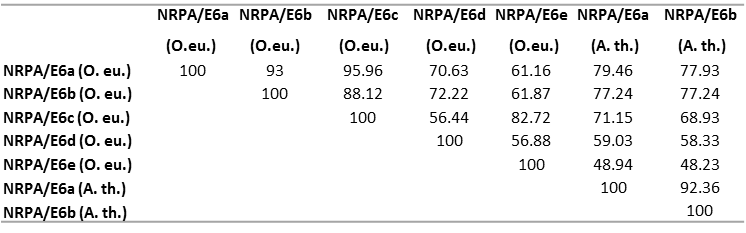


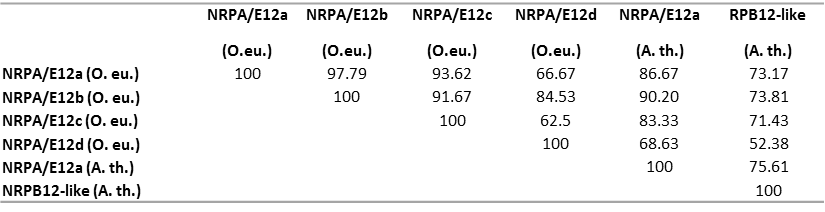


Figure S1

**Figure S1:** Amino acid identities between the identified olive cultivar “Picual”, NRP common subunits and Arabidopsis NRP common subunits. Sequence alignments were performed by Protein BlastP with standard parameters. O.eu.: olive”Picual” cultivar; A. th.: *Arabidopsis thaliana*.

NRPA/D5a ------------------------------------------------------------ 0

NRPA/D5b ------------------------------------------------------------ 0

NRPA/D5c ------------------------------------------------------------ 0

NRPA/D5(A.th.) ------------------------------------------------------------ 0

NRPB5a(Z.m.) ----------------------------------------------------------MS 2

NRPB5b(Z.m.) ------------------------------------------------------------ 0

NRPE5 -------------------------------------MTKEDGVDTDVDGE--SLGNCLS 21

NRPE5(A.th.) ---------------------------------------------MEVKGKETASVLCLS 15

NRPE5-Like(A.th.) ---------------------------------------------MEGKGKEI----VVG 11

NRPE(Z.m.) MDPHQFAPSQQSALTMESAEITAAAAAARAPNGAARAIVEDDD----EDDDVPEVAACIS 56

NRPA/D5a --MV-LSDEEIARLFRIRKTVMQMLRDRGYTVGDFEIDMSKHQFLSKYGENMKREDLIII 57

NRPA/D5b --MV-LSDEEITRLFRIRKTVMQMLRDRGYIVSDFEIDMSKHQFLSKYGENTKREDLVII 57

NRPA/D5c --MV-LSDEEITRLFRIRKTVMQMLRDRGYIVSDFEIDMSKHQFLSKYGENMKREDLVII 57

NRPA/D5(A.th.) ---M-LTEEELKRLYRIQKTLMQMLRDRGYFIADSELTMTKQQFIRKHGDNMKREDLVTL 56

NRPB5a(Z.m.) AGLV-TDEATVGRLYRIRRTVMQMLRDRGYLVVDHELATSRRDFLRKFGESFHREDLLIN 61

NRPB5b(Z.m.) --MA-SPDDEISRLFRIRRTVYEMLRDRGYGVRDEQIKLERHKFIERYGNPVRRDELTFN 57

NRPE5 GFID-DGSIESHRYYLARRTTLEMLRDRGFAVPNAEIEVSLQEFRNKHGQKPDIDNLRIT 80

NRPE5(A.th.) KYVD-LSSEESHRYYLARRNGLQMLRDRGYEVSDEDINLSLHDFRTVYGERPDVDRLRIS 74

NRPE5-Like(A.th.) HSIS-KSSVECHKYYLARRTTMEMLRDRGYDVSDEDINLSLQQFRALYGEHPDVDLLRIS 70

NRPE(Z.m.) TMLDRGGSVESHRLFLARRTALEMLRDRGYAVPEEELARTLPEFRAWWEYRPELERLAFS 116

. : : ::. :******: : : :: .* : *

NRPA/D5a KELRSNPSEKIYVFFPEEAKVGVKTMKT-YTNRMKDDDVHRAILVVQHNLTPFARTCISE 116

NRPA/D5b KDLRSNPSEKIYVFFPEEAKVGVKTMKT-YTNRMKDDDVHRAILVVQQNLTPFARTCISE 116

NRPA/D5c KDLRSNPSEKIYVFFPEEAKVGVKTMKT-YTNRMKDDDVHRAILVVQQNLTPFARTCISE 116

NRPA/D5(A.th.) KAKRNDNSDQLYIFFPDEAKVGVKTMKM-YTNRMKSENVFRAILVVQQNLTPFARTCISE 115

NRPB5a(Z.m.) KYKKNDPSDQIYVFFPNDDKVGMKHIKK-YVEMMTHENVSRAVLVLQQNLTPFAKSFLIE 120

NRPB5b(Z.m.) ATKLNGPSDQIYVFFPNEAKPGVKTIRN-YVEKMKNENVFAGILVVQQALSAFARSAVQE 116

NRPE5 SLHRDDPSIKVLVIFCGPQIVKVNVIRAIATQIVNRDTLSRLILVVQNKITNQALK-AVG 139

NRPE5(A.th.) ALHRSDSTKKVKIVFFGTSMVKVNAIRSVVADILSQETITGLILVLQNHVTNQALK-AIE 133

NRPE5-Like(A.th.) AKHRFDSSKKISVVFCGTGIVKVNAMRVIAADVLSRENITGLILVLQSHITNQALK-AVE 129

NRPE(Z.m.) TTLASDPSSKVKVVFCPPGPVKIAAIRLIYTEV-KDENLSRLILILQGKIMSTTRESIKE 175

. : :: :.* : :: .: . : : :*::* : :

NRPA/D5a ISTKFHLE--------------EAELLVNIKEHVLVPEHQLLTPEEKKSLLERYTVKETQ 162

NRPA/D5b ISTKFHLEVFQVIIDTFFTLLQEAELLVNIKEHVLVPEHQLLTPEEKKTLLERYTVKETQ 176

NRPA/D5c ISTKFHLEVF-----------QEAELLVNIKEHVLVPEHQLLTPEEKKTLLERYTVKETQ 165

NRPA/D5(A.th.) ISSKFHLEVFQ-----------EAEMLVNIKEHVLVPEHQVLTTEEKKTLLERYTVKETQ 164

NRPB5a(Z.m.) LEPKIHLEIFQ-----------EAEMLINIKEHVLVPEHQVLTNEEKKTLLERYTLKETQ 169

NRPB5b(Z.m.) VSQKYHLEVFQ-----------EAELLVNIKDHVLVPEHVLLTPEDKKTLLERYTVKETQ 165

NRPE5 LF-SFKVEIFQ-----------ITDLLVNITKHDCKPKHQVLTEQEKQSLLKKYSLNETQ 187

NRPE5(A.th.) LF-SFKVEIFQ-----------ITDLLVNITKHSLKPQHQVLNDEEKTTLLKKFSIEEKQ 181

NRPE5-Like(A.th.) LF-SFKVELFE-----------ITDLLVNVSKHVLRPKHQVLNDKEKESLLKKFSIEEKQ 177

NRPE(Z.m.) IF-RFKVDTFQ-----------ITELLVNITKHVLKPKHEVLTAEGKAKLLKEYNVVDSQ 223

: ::: :::*:*:..* *:* :*. : * .**:.:.: :.*

NRPA/D5a LPRIQVTDPISRYYGLKRGHVVKIIRPSETAGRYVTYRYVV 203

NRPA/D5b LPRIQVTDPIARYYGLKRGHVVKIIRPSETAGRYVTYRYVV 217

NRPA/D5c LPRIQVTDPIARYYGLKRGHVVKIIRPSETAGRYVTYRYVV 206

NRPA/D5(A.th.) LPRIQVTDPIARYFGLKRGQVVKIIRPSETAGRYVTYRYVV 205

NRPB5a(Z.m.) LPRIQITDPIARYYGLRRGQVVKIIRPSETAGRYVTYRYVV 210

NRPB5b(Z.m.) LPRIQITDPIARYYGMKRGQVVKITRASETAGRYITYRYVV 206

NRPE5 LPRMLQKDAIARYYGLEKGQVVKVMYSGELTQLHTSYRCVW 228

NRPE5(A.th.) LPRISKKDAIVRYYGLEKGQVVKVNYRGELTESHVAFRCVW 222

NRPE5-Like(A.th.) VSKFFGIWIITSTSSS---------------DSEPAFL--- 200

NRPE(Z.m.) LPRMLENDAVARYYGLGKGTVVKVIYDSELTGNHVTYRCIT 264

: :: : . ::

Figure S2

**Figure S2: Olive cultivar “Picual” NRPE5 subunit maintains the short N-terminal extension of the NRPE5 proteins.** NRPE5 subunits’ sequence alignments were performed by the Clustal Omega software with standard parameters. Red line indicates the N-terminal extension of the NRPE subunits. A. th.: Arabidopsis; Z.m..: *Zea mays*. Other NRP5 subunits correspond to olive cultivar “Picual”. ”*” : indicates perfect alignment ; ”:” : denotes the amino acids belonging to the group exhibiting strong similarity ; ”.” : represents a site belonging to the amino acids exhibiting slight similarity.

Figure S3

**Figure S3: Global mRNA levels in different organs and tissues.** Median and SD of the mRNA reads (RPKM) from the RNA-Seq analyses in different organs and tissues analyses {Ramirez-Tejero, 2020 #1906}. Total mRNA reads were normalized in relation to four different constitutively expressed genes: Act-19, L2-27, EF1a and Btub-28. *pvalue < 0.05 (Student’s t-test), except when comparing flowers to fruit.


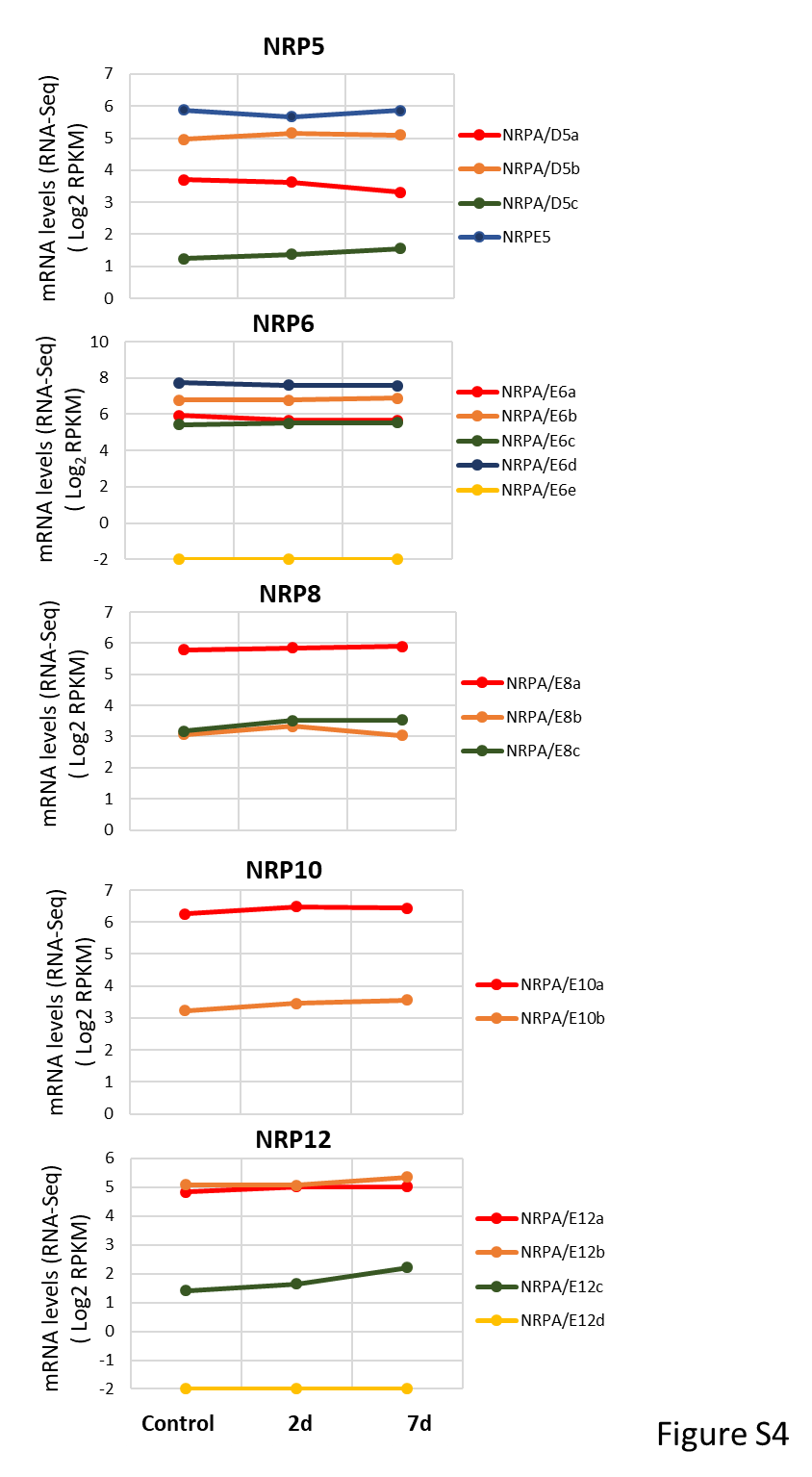


**Figure S4: RNA polymerase common subunit genes expression pattern during the *V. dahliae* early infection processin cultivar “Frantoio”.** Data from previously published RNA-Seq datasets ([Jiménez-Ruiz et al., 2017](#_ENREF_19)). Expression profile of the different NRP genes from olive cultivar “Frantoio” 1 and 7 days after *V. dahliae* infection. The control corresponds to the control group of non inoculated plants, handled in the same way as in the absence of the pathogen. Data correspond to reads per kilobase of transcript per million mapped reads (RPKM). Data are represented as Log_2_(RPKM). Values ≤ -2 are considered no expression and represented with a value of -2.
